# Co-design of ‘Ways of Being’, a web-based experience to optimise online arts and culture for mental health in young people

**DOI:** 10.1192/bjb.2023.102

**Published:** 2025-02

**Authors:** Rebecca J. Syed Sheriff, Eloise Sinclair, Jen Young, Sophia Bhamra, Louise Chandler, Tharuka Arachchige, Helen Adams, Laura Bonsaver, Evgenia Riga, Laura Bergin, Negin Mirtorabi, Leenah Abuelgasim, Hadassah Beuchner, John Geddes

**Affiliations:** Department of Psychiatry, University of Oxford, UK

**Keywords:** Psychosocial interventions, adolescent, depressive disorders, anxiety or fear-related disorders, web-based

## Abstract

**Aims and method:**

We aimed to co-design an intervention optimising the benefits of online arts and culture for mental health in young people for subsequent testing in a trial. Co-design followed the double diamond phases of design, discover, define, develop and deliver.

**Results:**

Navigating the views of all co-designers to produce a testable resource demanded in-depth understanding, and frequent iterations in multiple modalities of the theoretical basis of the intervention, amplification of youth voice and commitment to a common goal.

**Clinical implications:**

Co-design with a broad range of collaborators with a shared vision was valued by young co-designers and produced an effective intervention. Co-design allowed the theoretical basis to be followed and refined to create an engaging, practical and testable web experience, aiming to optimise the mental health benefits of online arts and culture for young people in a randomised controlled trial.

Three–quarters of mental disorders emerge before 25 years of age, yet young people are the least likely to receive appropriate care.^1,2^ Common mental disorders (CMDs) in young people, such as depression and anxiety, have long-term sequelae on mental and physical health, social support, relationships, levels of employment and health risk behaviours, including self-harm.^3,4^ There are significant direct and indirect costs over the life course,^2^ even when the young person's symptoms do not reach the diagnostic threshold for mental disorder at the time.

The provision of interventions by health services, such as antidepressant medication and/or talking therapies, are neither accessible nor acceptable to many young people.^5,6^ Health services focus on those with diagnosed conditions, but many young people do not realise they are experiencing poor mental health or fear stigma and struggle to get any help.^6,7^ The National Health Service has long waiting lists for young people (especially child and adolescent mental health services for those aged under 18 years) identified for specialist referral for their mental health. Underrepresented young people, such as ethnic minorities^8^ and those who identify as LGBTQ+,^9^ have high rates of mental health problems, low rates of help-seeking^8^ and are particularly poorly represented in mental health research.^10^ In addition, although there is increasing interest in community assets in improving health, there is currently a lack of evidence for these resources for CMDs in young people,^11,12^ and young people are feeling increasingly disconnected from their communities.^13^ There is also a lack of engagement from young people in determining the most fruitful approaches to support their mental health.^11^ Therefore, identifying effective methods of engaging young people in developing mental health interventions is a priority.

In the UK, young adults spend more time online than any other age segment. The 2022 Ofcom report states that young adults continue to spend the most time online, with 18- to 24-year-olds spending an average of 5 h 6 min online a day, more than an hour longer than the UK average.^14^

Engagement with arts and culture is associated with health benefits.^15^ The mechanisms thought to improve mental health include emotional activation, aesthetic engagement, interaction, social and cognitive stimulation, sensory activation and imagination.^16–18^ Online arts and culture (OAC) may reach these subclinical and/or difficult to engage groups.

Experimental evidence assessing OAC for CMDs in young people is lacking, and there is an increasing emphasis on the need to develop remote resources and digital interventions to aid with mental health in view of the increasing rates of CMDs both before and in the wake of the COVID-19 pandemic.^19–21^ However, little empirical evidence exists to support OAC with regard to mental health in young people.^22–24^

Intervention development guidelines have been criticised for overemphasising the theoretical basis of intervention development that requires vast technical skills and resources.^25^ Some have highlighted the importance of common sense, judgement and logic during intervention development.^26^ Most guidance for intervention development recommends some element of co-production^25,27^ with the intended end-users of the product, to aid feasibility and implementation.

## Aims

We aimed to co-design an intervention optimising the mental health benefits of OAC for mental health for subsequent testing on depression and anxiety in a trial. We aimed to rapidly develop an intervention that was systematic, grounded in theory, deliverable and testable at low cost. A co-design approach was taken on the basis of a lack of previous literature on the components and mechanisms for OAC for mental health in young people, the tight timescale in the context of escalating CMDs in young people and the low level of resourcing. Because of COVID-19 restrictions in place during this study, the co-design process was carried out online. The aim of this paper more specifically is to describe our approach to the co-design process and how it was experienced in detail, as this has been a shortfall in previous participatory research with young people.^28^

## Method

**Fig. 1:**
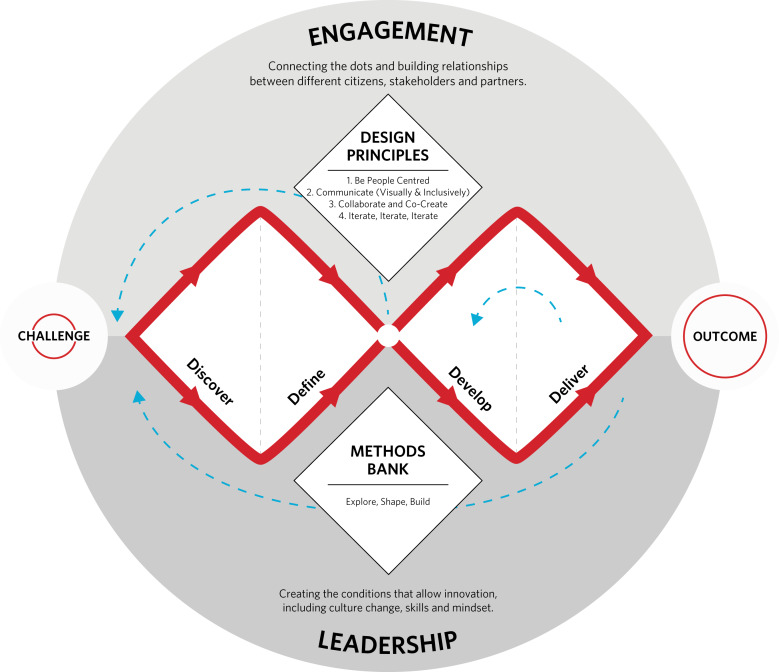
Design Council Double Diamond principles framework.

To efficiently use resources and enable remote working and collaboration, we planned intervention development to be user-focused from the outset, dynamic, iterative, creative, flexible and forward-looking to evaluation and implementation.^29^ To do this, we planned to co-design an intervention according to the Design Council Double Diamond principles of discover, define, develop and deliver (Fig. 1). All research involving human participants was granted ethical approval by the University of Oxford Central University Research Ethics Committee for this series of studies before the commencement of each study (approval reference numbers R70187/RE001-7).

### Discover

The discovery phase comprised a cross-sectional survey, systematic review and public patient involvement (PPI) consultation to ascertain the views of service users regarding the direction of research. The cross-sectional survey was conducted in mid-2020 to gain a deeper understanding of who used online cultural resources and the perceived benefits and impact on mental health. For the purposes of this study, OAC was defined as online resources from cultural institutions, including museums, theatres, art galleries, libraries, archives and natural heritage organisations.

We recruited 1056 participants aged ≥16 years for the initial survey between 17 June and 22 July 2020. Participants were recruited through Facebook adverts, a press release, a pop-up advert that appeared on the Ashmolean Museum website, the Ashmolean Museum's public relations avenues (e.g. Twitter and a newsletter) and student unions. The methods are described in more detail elsewhere.^30^

In addition, we conducted a systematic review aiming to evaluate the acceptability and effectiveness of community assets for mental health. This included interventions delivered at and/or by museums and art galleries to adults and young people, either in person or online. The protocol of this review is publicly available,^31^ as is the final review.^12^

We talked to three people who had used mental health services from Oxfordshire and Blackpool, to explore their views on researching OAC for mental health, particularly eliciting their views as to whether they thought currently available online arts and cultural resources could be useful for mental health. This involved exploration of whether they thought any avenues for research were particularly promising, including how, why and for whom they thought it could be useful.

To define the perceived mental health benefits of OAC for young people, individual semi-structured interviews were conducted through August and September 2020 with 13 participants aged 16–24 years. Participants were diverse in sociodemographic characteristics, and varied in their use of OAC and in their mental health status. Rich interview data were analysed using a reflective thematic analysis.

### Define

A stakeholder workshop was held on the 13 August 2020, involving a wide group of stakeholders that was also split into smaller mixed breakout groups for further informal discussion and creative activities. Stakeholders comprised PPI, young people, multidisciplinary researchers (from internet research, psychology, psychiatry), youth engagement, and museum and culture professionals and designers (see Table 1). The workshop involved the presentation of findings of the discovery phase and recent epidemiological studies on mental health, followed by breakout groups for creative activities to interrogate the evidence and allow for experiential reflection. This enabled workshop participants to discuss potential target groups and contexts, and guide intervention development in light of the findings of the discovery phase.

**Table 1:** Description of stakeholders

Number of people	Role
7	Multidisciplinary O-ACE core team, including researchers and staff from the Department of Psychiatry at the University of Oxford, Oxford Internet Institute and Oxford University Gardens, Libraries and Museums (GLAM)
3	Lived experience experts
3	Lived experience experts from within the target age range (16–24 years)
1	Experimental medicine researcher (Department of Psychiatry, University of Oxford)
1	Child and adolescent psychology researcher
1	Young people engagement officer (GLAM)
1	Ashmolean Head of Public Engagement
1	Ashmolean Head of Online Engagement
1	Managing director of the creative digital agency

### Develop

We developed a core team of co-designers that evolved throughout the co-design process according to need. Researchers were based in Oxford (Oxford University Gardens, Libraries and Museums; Department of Psychiatry at University of Oxford; and Oxford Internet Institute), PPI were from Buckinghamshire and the young co-designers were from all over the UK and overseas. The 12 co-designers were a diverse group of young people who were recruited from youth PPI groups, volunteers from university staff and students, and included some young people involved in the qualitative interview study who expressed an interest in continued involvement in the project. All young co-designers were reimbursed financially for their time (in workshops and giving feedback) at an agreed hourly rate. Throughout the development phase we allowed the co-design team to evolve according to the design need.

#### Ethics approval statement

The University of Oxford Research Ethics Committee granted ethical approval for this study before its commencement (reference number R70187/RE006).

Development of the intervention occurred over a 3-month period (September to November 2020) in an iterative process. Throughout, weekly meetings were attended with Imagineear (www.imagineear.com), a production company experienced in content creation and platform provision for museums and art galleries, as well as health services.

**Fig. 2:**
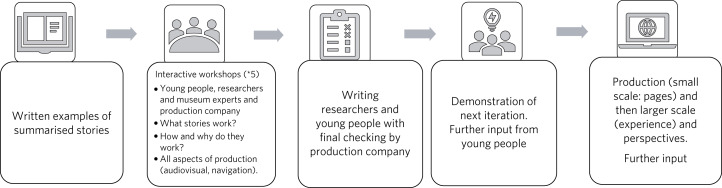
Overview of the development phase.

Youth voice took precedence. We conducted five workshops with young people and encouraged constant feedback in a number of modalities (messaging, email, telephone calls) in an iterative process involving small- and large-scale production, co-production sprints and refinement. Stages of intervention development included the presentation and selection of stories, generation of viewpoints, determination of visual design and audio-visual preferences, and navigation and optimisation of the web experience (see Fig 2).

### Deliver

Diverse young people actively contributed to the design of a randomised controlled trial (RCT) to evaluate the co-designed intervention for depression and anxiety in young people.^32^ The evaluation included qualitative and quantitative methodologies, and is described in more detail elsewhere.^33^

## Results

### Discover

The cross-sectional survey revealed information relevant to the design of an online intervention; for example, the self-reported mental health benefits of online culture were associated with regularity of use of OAC and differed with age. Although people aged 16–24 years reported potential mental health benefits of OAC, they were less likely to be regular users of it. Echoing population studies, higher proportions of people aged 16–24 years had levels of distress, signifying probable anxiety and/or depression. High proportions of the respondents of the survey were female, White, university educated, older and on a high income; however, the 77 younger people (aged 16–24 years) who responded to the survey had more diverse sociodemographic characteristics than the older age groups.

The systematic review^12^ demonstrated that studies of arts and culture interventions mainly involved art therapies, particularly in elderly populations and those with physical health conditions. However, there was a scarcity of experimental studies on the effect of receptive engagement with the arts and culture on CMDs in young people, either in person or online.

PPI participants from Oxfordshire and Blackpool mental health services described that they had not previously thought of OAC as a potential mental health intervention and had not used OAC themselves because of a lack of awareness of it, but could see its potential as a source of mental health support. They thought that OAC could be a useful line of intervention research in supporting mental health, but cautioned that accessibility needed to be improved, particularly for those who experience mental ill health. They highlighted that to be useful for mental health, content should be accessible, diverse, engaging, interactive and inspire exploration and human connection. Although the PPI participants were over 24 years of age, they also thought that it could be of particular use for adolescents and young adults.

### Define

The stakeholder workshop demonstrated a general enthusiasm for OAC for mental health and its potential use in populations with low levels of conventional help-seeking. Ways in which OAC was perceived as being helpful for mental health were through absorption, exploration and discovery. A common theme from the breakout groups were the benefits of going ‘down a rabbit hole’ and becoming absorbed in exploring interconnected content.

The outcome of the stakeholder workshop was to target young people (aged 16–24 years), given the increasing rates of anxiety and depression, low levels of help-seeking, poor mental health literacy and preference of creative ways of supporting mental health in this population. We then embarked on a qualitative interview study to identify the potential utility of OAC for CMDs in young people aged 16–24 years, in which there were 13 participants. Participants identified that culture and the arts were a potentially useful approach to supporting their mental health, and that benefits were more likely to be derived with familiarity and regular use. Participants described some advantages of online versus in-person engagement, including being able to use it regularly, remotely and on demand, as well as the capability for online engagement to provide deeper and broader commentary and more diverse content. In particular, human stories were identified as the single most important ingredient for mental health benefit. Participants recommended more content describing the experiences of a range of people from different backgrounds that they could connect with on a human level, and more alternative viewpoints.

### Develop

#### Initial considerations

Young co-designers gave a clear idea of how and when they might use the online tool, and preferred a desktop/laptop rather than a mobile app, to allow for an expansive design. Some were also wary of their own overuse of their mobile telephone. Around halfway through the design, the young co-designers settled on ‘Ways of Being’ as the name for the new tool.

#### Theoretical framework

**Fig. 3:**
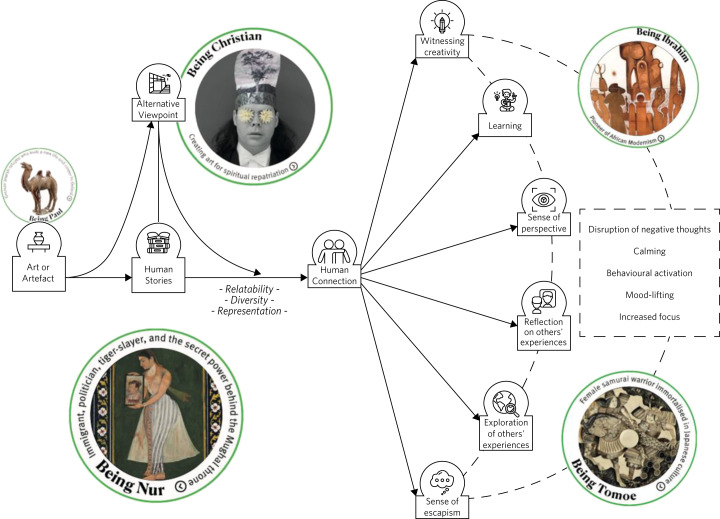
Conceptual framework of Ways of Being, including imagery. © Ashmolean Museum, University of Oxford and courtesy of the artists.

A conceptual framework of the mental health benefits of OAC, based on theory generated in the qualitative interview study, was continually developed and refined in an iterative process informed by young co-designers (see Fig. 3).

The underlying theory was explained in a variety of ways, using different modalities to different members of the team, young co-designers, researchers and the creative digital agency (Imagineear) with whom we were working (www.imagineear.com).

Figure 3 is a schematic describing visually findings from the qualitative study,^34^ with additional insights from the process of co-design. It shows that the ingredients of OAC perceived as being most likely to benefit mental health were diverse human stories about the personal challenges of real people behind the art and artefacts, and a variety of viewpoints with which they could connect on a human level. The potential mechanisms of impact on mental health were bringing a sense of perspective and providing opportunities for reflection, learning, escapism, creativity, exploration and discovery. Perceived impacts on mental health were the disruption of negative thought patterns, lifting mood, and increased feelings of calm and proactivity.

#### Selection of stories

The first step in co-design was to find a method of choosing which art or artefact had a human story associated with it that would have potential mental health benefits. We engaged museum curators to uncover new angles and stories related to objects, with an emphasis on the diversity of the associated human elements. Thumbnails of potential stories were presented at workshops, and feedback was encouraged in multiple modalities to refine, reject or suggest new ideas. During workshops, young co-designers would say that certain story ideas ‘resonated’. There tended to be a high degree of agreement between the young co-designers. These would be the stories that they then chose to develop for Ways of Being. This was a process that then became known as ‘resonance testing’ within the co-design team. Stories of the lives of people who faced challenges often resonated strongly with young co-designers, with a preference for those whose stories were not widely known, including women and those from non-Western cultures that were less well-represented in a typical European art museum.

Building on initial feedback that we needed to include artworks and stories reflecting diversity and difficult themes still relevant today, some stories from antiquity were presented. However, young co-designers could not see the links to their own lives. On deeper examination, it appeared that in most cases, the archaeological record simply did not offer sufficient personalised detail about everyday lives to engage the young person.

#### Generation of viewpoints

Young co-designers wanted a way for users to share perspectives in a positive way. The pressure and judgement perceived with social media was a significant concern for the young co-designers, and they were cautious of a commentary feature similar to YouTube. However, arts and culture were seen as an open, safe space where different opinions were acceptable. Co-designers felt that a comments function could help facilitate opinion and interpretation sharing, as long as it was adequately monitored, and users could decide on levels of anonymisation. A comments function was enabled on every page, which was linked to an email address where a moderator could publish these to the comments tool so that participants could see their comments alongside others.

#### Determination of audio-visual preferences

Young co-designers had a preference for an audio option, with a familiar, friendly and authentic voice to guide the user through the core stories. In contrast with Imagineear's established samples of voice actors – because of the need for professional voice actors in production to ensure technical quality – young people consistently felt that these were too ‘polished’ to connect with, and had a clear preference for a ‘real and authentic voice’. This was the most contentious debate throughout the development phase. This was finally resolved by allowing the young co-designers to independently vote on >40 samples of voices. The voice with the highest number of votes, which was chosen to be the ‘guiding voice’, was the only voice that was not that of a professional voice actor, but rather the anonymised voice of one of the researchers. The voices with the next highest number of votes (one male and one female) provided the voices for the in-depth stories.

Young co-designers found too much intense white background was overly clinical, ‘headache inducing’ and too redolent of educational resources. Subsequently, the design of each ‘core story’ developed a colour palette that matched colours from the artworks and objects associated with that person's story.

#### Navigation

Of paramount importance to co-designers was a welcoming feel and easy and consistent navigation. A difficult balance was how to structure content: although a non-linear layout was most transparent and gave more optionality about ‘where to start’, too little structure could be overwhelming, confusing and frustrating. Another preference was to emulate the feel of immersion that can be felt in a gallery space, being surrounded by things and being able to pick what to look at, to give a sense of user autonomy, exploration and discovery.

Through an iterative process of designing the format for the tool, a core story was surrounded by in-depth stories that expanded on key parts of the story and other material (such as more examples of their artwork). There were 11 ‘core-stories’. Each core story and related content formed a ‘constellation’, and constellations were interconnected through content that contained similar themes. There was a clear desire to read/listen to the core story and explore related content with ‘no dead ends’. Through various iterations, a framework was developed that supported participants to read only, read and listen, view images and listen, and for those who wanted a blend of everything, automated multimedia slideshows were co-designed.

The final web experience was designed to be entered at a time and place convenient to the individual on their own device. Introductory pages illustrated the features of Ways of Being. A non-hierarchical menu page gave the choice of 11 diverse ‘stories’, each of which encompassed a visual and a title, e.g. ‘Being Ibrahim’. Once entered, these led into a long-form story (which could be listened to or read) interspersed with visual media, and then further ‘deeper’ stories that were interconnected and presented in a paginated style to prevent the fatigue of constant scrolling. Alongside the stories was a comments tool that enabled users to add their own viewpoint to a variety of viewpoints prepopulated during co-production, enabling young people to express their responses anonymously. These viewpoints were then redisplayed within Ways of Being.

### How co-design was experienced

As young co-designers from outside of the research institution, we felt listened to throughout the processes, and the final prototype for testing in an RCT reflected the developing conceptual framework as well as our views and preferences for how to engage with and use the web experience. We found the methods of engagement flexible and responsive to our preferences. For example, the interactive workshops were digital, and we could speak, use the chat function and/or use email between workshops. This was a valuable experience that added to our skills and experience that are applicable elsewhere. Overall, we felt that we were contributing in a meaningful way to something that had the potential to have a positive impact on many others.

## Discussion

Navigating the views of all co-designers to produce a testable resource demanded in-depth understanding, and frequent iterations in multiple modalities of the theoretical basis of the intervention, amplification of youth voice and commitment to a common goal. We generated methods to develop a novel intervention, following and developing the theoretical basis of the findings of the discovery phase, rather than adapting a pre-existing intervention. This had the advantage of tailoring the intervention to a specific age range (with unique challenges), but involved navigating emerging methodological and theoretical processes. One of the reasons cited that there has been such variation in the quality of the reporting of participatory research such as co-design previously, maybe due to its dual purpose as a research methodology and as an approach that enables action and change.^28^

### The process

Participatory research may empower participants, increase health literacy and embed research into the community.^35^ Most participatory research with young people takes place in schools or healthcare settings.^28^ Museums and other cultural institutions offer an alternative setting where interventions can take place, either on-site or online, and encourage freedom of expression. Such community settings may be more acceptable for young people,^5^ but more needs to be done to make these venues attractive and relevant to young people – those aged 16–24 years are the most underrepresented age group among museum visitors in the UK. Young people, seldom-heard communities and minority groups are likely to benefit most from co-produced interventions because they are traditionally not well-represented in the design and evaluation process.^36^

The Ways of Being intervention was subsequently tested and shown to have effectiveness compared with a high-quality museum website (the Ashmolean Museum) in an RCT.^33^ However, there is a lack of evidence base for the benefits of co-produced health interventions more generally. Most co-produced interventions are not scientifically evaluated nor compared with a non-co-produced control.^37^ We have demonstrated that co-production is feasible and had high levels of engagement with young co-designers, with many wishing to stay engaged with the study after its official close.

### Developing Ways of Being

During the co-design process, we identified areas of input that led to the final intervention being markedly different to a typical museum website. The overarching design was to maximise the feeling of human connection. For example, even where audio was implemented, young people preferred non-professional voices to guide the story over professional voiceovers, to enhance the sense that it was a ‘real’ person speaking.

In addition, for young people there seemed to be value in ‘looking out’ at shared human struggles via the intervention. In contrast, many culture-based interventions and activities aimed at adults focus on using art to access one's own emotions, such as mindfulness sessions. There was a clear preference for diversity of content that was also demonstrated in the theoretical framework for mental health benefit. However, there was an appreciation that many cultural institutions lack this diversity of content, which was perceived as potentially detrimental.

### Implications

At present, there are no well-recognised guidelines for evidence-based co-production methodologies, which can be time-consuming and intensive for all parties and therefore need careful planning. Nor is there guidance on when co-production may not be appropriate.^38^ Future research should identify the necessary components for co-production, as well as outlining important practical and ethical considerations.

Co-design allowed the theoretical basis to be followed and refined to create an engaging, practical and testable web experience aiming to optimise the mental health benefits of online culture for young people in an RCT.

Our work adds to the evidence base supporting community interventions for mental health, which may reach a wider population than traditional health interventions. The co-design method could be used across areas of public health, as well as with other modalities of community resource, but more research is needed as to where these methodologies are most effectively utilised and any possible harms.

## Data Availability

The data that support the findings of this study are available from the corresponding author (R.J.S.S.), upon reasonable request.

## Biographies

**Rebecca J. Syed Sheriff,** Department of Psychiatry, University of Oxford, UK; **Eloise Sinclair,** O-ACE co-design group, Department of Psychiatry, University of Oxford, UK; **Jen Young,** O-ACE co-design group, Department of Psychiatry, University of Oxford, UK; **Sophia Bhamra,** O-ACE co-design group, Department of Psychiatry, University of Oxford, UK; **Louise Chandler,** O-ACE co-design group, Department of Psychiatry, University of Oxford, UK; **Tharuka Arachchige,** O-ACE co-design group, Department of Psychiatry, University of Oxford, UK; **Helen Adams,** GLAM, University of Oxford, UK; **Laura Bonsaver,** GLAM, University of Oxford, UK; **Evgenia Riga,** Department of Psychiatry, University of Oxford, UK; **Laura Bergin,** School of Anthropology, University of Oxford, UK; **Negin Mirtorabi,** O-ACE co-design group, Department of Psychiatry, University of Oxford, UK; **Leenah Abuelgasim,** O-ACE co-design group, Department of Psychiatry, University of Oxford, UK; **Hadassah Beuchner,** O-ACE co-design group, Department of Psychiatry, University of Oxford, UK; **John Geddes,** Department of Psychiatry, University of Oxford, UK.
